# A Developmental Study of Abnormal Behaviors and Altered GABAergic Signaling in the VPA-Treated Rat Model of Autism

**DOI:** 10.3389/fnbeh.2018.00182

**Published:** 2018-08-21

**Authors:** Qianling Hou, Yan Wang, Yingbo Li, Di Chen, Feng Yang, Shali Wang

**Affiliations:** ^1^Cerebrovascular Disease Laboratory, Institute of Neuroscience, Chongqing Medical University, Chongqing, China; ^2^Department of Physiology, School of Basic Medical Sciences, Chongqing Medical University, Chongqing, China; ^3^Lieber Institute for Brain Development, Johns Hopkins University Medical Center, Baltimore, MD, United States

**Keywords:** autism, GABA, VPA, GAD67, autistic-like behaviors, motor coordination, spatial learning and memory

## Abstract

Although studies have investigated the role of gamma-aminobutyric acid (GABA)ergic signaling in rodent neural development and behaviors relevant to autism, behavioral ontogeny, as underlain by the changes in GABAergic system, is poorly characterized in different brain regions. Here, we employed a valproic acid (VPA) rat model of autism to investigate the autism-like behaviors and GABAergic glutamic acid decarboxylase 67 (GAD67) expression underlying these altered behaviors in multiple brain areas at different developmental stages from birth to adulthood. We found that VPA-treated rats exhibited behavioral abnormalities relevant to autism, including delayed nervous reflex development, altered motor coordination, delayed sensory development, autistic-like and anxiety behaviors and impaired spatial learning and memory. We also found that VPA rats had the decreased expression of GAD67 in the hippocampus (HC) and cerebellum from childhood to adulthood, while decreased GAD67 expression of the temporal cortex (TC) was only observed in adulthood. Conversely, GAD67 expression was increased in the prefrontal cortex (PFC) from adolescence to adulthood. The dysregulated GAD67 expression could alter the excitatory-inhibitory balance in the cerebral cortex, HC and cerebellum. Our findings indicate an impaired GABAergic system could be a major etiological factor occurring in the cerebral cortex, HC and cerebellum of human cases of autism, which suggests enhancement of GABA signaling would be a promising therapeutic target for its treatment.

## Introduction

Autism, or autism spectrum disorder (ASD), is a neurodevelopmental condition as diagnosed by the presence of behavioral impairments in three main domains including impaired social interaction, compromised communication and restrictive stereotyped behaviors and interests (Baron-Cohen et al., 1985; Frith and Happé, 2005; American-Psychiatric-Association, 2013; de la Torre-Ubieta et al., 2016). Autism is also associated with clinical comorbidities including anxiety and cognitive deficits (Lecavalier, 2006). Children with autism can experience anxiety more intensely and more often than normal children; and thus, they can have trouble with understanding and communicating emotions. Although a subpopulation of patients with ASD are defined as high functioning individuals that display superior abilities in very specific and focused cognitive domains including enhanced pitch processing and memory, increased pattern discrimination and superior spatial performance, the vast majority of individuals suffering from ASD exhibit mental retardation whose cognition can vary considerably from severely mentally impaired to markedly above average (Caron et al., 2004; Frith and Happé, 2005; Minshew and Williams, 2007; Crespi, 2016).

Many brain regions in autism show abnormalities including loss of CA1 and CA3 pyramidal neurons as well as granule cells in the hippocampus (HC; Raymond et al., 1996; Gao et al., 2016), decreased Purkinje cell counts and atrophy of the cerebellum (Ritvo et al., 1986; Ingram et al., 2000; Mostofsky et al., 2000; Sabaratnam, 2000), changes in temporal lobe physiology (Chi and Snyder, 2014), delayed sensorimotor development (Bernabei et al., 2003) and decreased gamma-aminobutyric acid (GABA)ergic chandelier and basket interneurons and altered local synaptic connectivity in the prefrontal cortex (PFC; Hashemi et al., 2017; Ariza et al., 2018; Liska et al., 2018). These abnormalities may contribute to the observed autism-spectrum behaviors and cognitive impairments. The 67 and 65 kDa isoforms of glutamic acid decarboxylase (GAD), the key enzymes for GABA biosynthesis, are expressed at altered levels in post-mortem brains of subjects diagnosed with autism, particularly decreased GAD67 mRNA levels in multiple brain regions including the PFC, temporal cortex (TC) and cerebellum (Akbarian and Huang, 2006; Yip et al., 2007). Emerging evidence indicates that an imbalance between excitatory and inhibitory synaptic transmission within neural circuits plays a role in autism, anxiety, ADHD and impaired spatial memory (Rubenstein and Merzenich, 2003; Gogolla et al., 2009; Chao et al., 2010; Edden et al., 2012; Han et al., 2012; Banerjee et al., 2013; Tyzio et al., 2014; Lee et al., 2017; Li et al., 2017). GABAergic system dysregulation, including decreased number of GABAergic interneurons and impaired GABAergic neurotransmission, has been proposed as a source for the modified balance of excitation-inhibition in the cerebral cortex, HC and cerebellum seen in clinical cases of autism (Han et al., 2012; Hashemi et al., 2017; Ariza et al., 2018). Excitation and inhibition imbalance are also commonly observed in rodent models of autism, and their correction by pharmacological interventions normalizes key autistic-like phenotypes in these animals (Han et al., 2012; Banerjee et al., 2013; Lee et al., 2017). Overall, these results suggest that an excitation and inhibition imbalance within the brain’s synapses may contribute to the development and maintenance of ASD.

A number of studies have shown that ASD is caused by a complex interaction of genetic and environmental factors (Sullivan et al., 2012; Favre et al., 2013; Yuen et al., 2015). While many studies have indicated that genetic factors play a key role in at least half of the cases of autism (Voineagu et al., 2011; Chahrour et al., 2012; Neale et al., 2012; Yu et al., 2013; Stefansson et al., 2014; Won et al., 2016), a wide range of potential environmental factors are thought to also contribute to development of autism (Nanson, 1992; Kinney et al., 2008; Angelidou et al., 2012; Lauber et al., 2016; Modabbernia et al., 2017). Thus, generation of well-defined animal models that can display core symptoms, neuropathological and behavioral features are essential for autism research (Gadad et al., 2013; Mandy and Lai, 2016). Valproic acid (VPA) is a drug commonly prescribed for patients with epilepsy and bipolar disorder (McElroy et al., 1989). However, VPA is also a potent teratogen and prenatal exposure increases the risk of giving birth to an autistic child (Nadebaum et al., 2011). In rodents *in utero* exposure to VPA also causes neurodevelopmental abnormalities found in humans, mimicking many of the features of ASD including increased repetitive behaviors, reduced social interaction, hypersensitivity and increased male vulnerability (Schneider and Przewlocki, 2005; Kim et al., 2011, 2013; Mehta et al., 2011; Olexová et al., 2016). These behaviors in rodents have led to the proposal that rats treated prenatally with VPA would function as an animal model for autism study.

Although many studies have investigated the role of GABAergic signaling in rodent neural development and behaviors relevant to autism (Tabuchi et al., 2007; Cusmano and Mong, 2014; Lauber et al., 2016; Olexová et al., 2016; Wei et al., 2016; Chau et al., 2017; Watanabe et al., 2017), the behavioral ontogeny as determined by changes in GABAergic system of different brain regions is poorly characterized. Here, we employed a VPA rat model of autism to investigate the autism-like behaviors and GABAergic GAD67 expression underlying these altered behaviors in multiple brain areas at the developmental stages from birth to adulthood. We found that VPA-treated rats exhibited behavioral abnormalities relevant to autism, including delayed nervous reflex development, altered motor coordination, delayed sensory development, autistic-like and anxiety behaviors and impaired spatial learning and memory. Prenatal VPA exposure dysregulated GAD67 expression and could alter the balance of excitatory-inhibitory neurotransmission in the cerebral cortex, HC and cerebellum. Our findings indicate that an imbalance of excitatory-inhibitory neurotransmission could be a major etiological factor occurring in the cerebral cortex, HC and cerebellum in human cases of autism, which suggests enhancement GABA signaling would be a promising drug target for its treatment.

## Materials and Methods

All experimental procedures were performed under the guidelines of the Experimental Laboratory Animal Committee of Chongqing Medical University and were in strict accordance with the principles and guidelines of the USA National Institutes of Health Guide for the Care and Use of Laboratory Animals (NIH Publications No. 80-23). The animal protocols used in this work were evaluated and approved by the Experimental Laboratory Animal Committee of Chongqing Medical University (Protocol 2015-051).

### Animals

**Figure 1:**
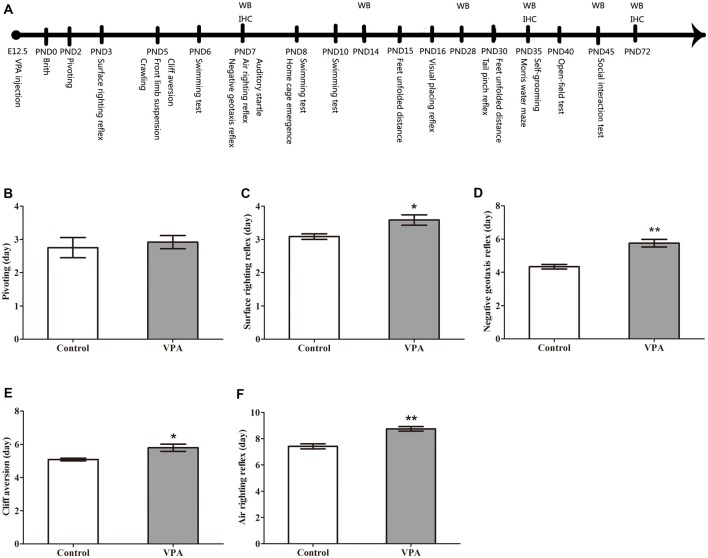
Valproic acid (VPA)-treated rats exhibited delayed nervous reflex development. **(A)** Experimental approach. A representative timeline of the behavioral tests, western blot (WB) and immunohistochemistry (IHC). **(B)** VPA-treated rats showed normal performance in the first pivoting appearance day compared to control rats (*p* > 0.05). **(C–F)** VPA-treated rats exhibited significantly longer latencies in behavioral ontogeny than control rats, including the first appearance day of surface righting reflex (*p < 0.05), negative geotaxis reflex (**p < 0.01), cliff aversion (*p < 0.05) and air righting reflex (**p < 0.01). Data are represented as mean ± SEM (*p < 0.05, **p < 0.01).

Experimentally naïve male and female Sprague-Dawley rats were purchased from the Animal Core Facility of Chongqing Medical University (Chongqing, China), and male and female animals were fed separately and housed in groups of 3–4. All rats were maintained under standard laboratory conditions at 22 ± 2°C, with 50 ± 10% relative humidity and on a 12 h-light/dark cycle, with food and water made available *ad libitum*. Female rats weighing 250–280 g and male rats weighing 280–350 g were used. Before any experimental procedures were carried out, animals were acclimated for 1 week to the experimental rooms. Their fertility cycle was controlled, and, when the females were in a pro-estrus state, the animals were allowed to mate overnight. Vaginal smears on glass slides were examined on the following morning; and if spermatozoa were found, it was designated as first day of pregnancy. Each pregnant rat was then housed separately and divided into control and VPA-treated groups. Valproate acid (Sigma-Aldrich) was dissolved in 0.9% saline at a concentration of 250 mg/ml. Females received a single intraperitoneal injection of 600 mg/kg sodium valproate (VPA) on E12.5 day after conception, and control females were injected with the same amount of physiological saline at the same time period. Females were housed individually and allowed to raise their own litters. The number of viable offspring born in both groups control and VPA-treated was normal. All animals exposed to VPA during gestation developed a characteristic “kink” in their tails, which was easily distinguishable from the aged-matched controls. The offspring were weaned on postnatal day (PND) 21. All subsequent experiments were performed only on the male offspring. Behavioral tests, western blot (WB) and immunohistochemistry (IHC) were performed on PND 2–72 (Figure 1A).

Since animal’s behavioral ontogeny was investigated, the same rats were applied to perform different simple behavioral tests at the different time points except Morris water maze. It made to be an unbiased and consistent to analyze the same animal behavioral ontogeny in this study. Morris water maze was carried out for seven consecutive days and it was more challengeable for animal to complete this task than that others. Therefore, independent rats were carried out for Morris water maze. It should be mentioned here that all experiment in WB and IHC was performed from independent animals who were not tested in the behavioral studies, which would exclude the effects of performed behaviors on GAD67 expression in the brain. Totally, 28 litters in control and 33 litters in VPA-treated group had been used in this study, respectively.

### Behavioral Tests

#### Nervous Reflex

##### Pivoting

On PND2, the pups were placed on a board and the time they spent to pivot the body (at least rotate 90°) was observed. The first day that the pups appeared to begin to successfully rotate 90° was recorded.

##### Surface Righting Reflex

Beginning on PND3, all newborns were placed in supine position on a board, and the time it took the pups to turn over to prone position and to stand on four paws in contact with the board was recorded. The first appearance day of surface righting reflex was defined as pups began to stand on four feet in 4 s.

##### Negative Geotaxis Reflex

The negative geotaxis reflex was observed beginning on PND7. Pups were placed to face down on a rough slope having a 30° incline and held there for 5 s, then and released to see whether they were able to turn face upwards (rotate 180°) in 30 s. The first day the animals appeared to successfully accomplish the task was recorded.

##### Cliff Aversion

Cliff aversion was evaluated beginning on PND5. Pups were placed on the edge of a box (30 cm high), with their forepaws and snout positioned over the edge. The first day when pups appeared to be able to return their bodies back to 1.5 cm from the “cliff” in 10 s was recorded.

##### Air Righting Reflex

Pups were dropped from supine position onto a bed of shavings from a height of 20 cm. The first day that they appeared to begin to land on four feet was recorded. The test was started on PND7 and repeated until pups were able to land on three or four feet for three consecutive times.

### Motor Tests

#### Front Limb Suspension

Forelimb grip was measured to test the forelimb strength beginning on PND5. Pups were allowed to grasp a wire strung across a stable object (30 cm high) and to hang onto the wire with both forepaws, and the time before they dropped was recorded.

#### Feet Unfolded Distance

On PND15 and PND30, the feet unfolded distance was measured. Each posterior limb was coated with red inkpad. Pups were dropped onto a sheet of white paper from a height of 30 cm to stimulate a natural falling response of landing with their feet unfolded, and the distance between two footprints was measured.

#### Crawling

Beginning on PND5, pups were placed on a board and the time it took the animals to display a linear crawling behavior was recorded. Clockwise or counterclockwise circling or spinning around with one arm fixed were discounted as crawling behaviors. The day of the first appearance crawling behavior was defined as pups being able to crawl in a straight line over a 3 cm distance.

#### Swimming Test

On PND6, PND8 and PND10, pups were put in warm water (25 ± 3°C) and their sport swimming coordination ability was observed. Performance of the pups was scored on a scale of 0–9, as based on points given for the angle maintained between the water and head, body use and swimming direction.

### Sensory Function

#### Auditory Startle

We began analysis of auditory startle response of the pups on PND7 and presented a loud click sound directly at a distance of 25–30 cm over the animals to assess whether or not a startle motion had occurred. A positive startle response was observed when the pups exhibited a “jerking” movement away from the sound source. The first day when the pup obviously displayed a “jerking” movement was recorded.

#### Home Cage Emergence

On PND8, pups were placed on the center of a box fresh shavings on one side of the floor and shavings from its home cage placed on other side. The time the pups spent to reach the shavings from its home cage while standing on four feet was recorded.

#### Visual Placing Reflex

Beginning on PND16, pups were gently suspended by the tail, and lowered towards a solid surface to observe whether they raised their heads and extended their forelimbs in a foot placement fashion. The first day when this reflex appeared to occur was observed.

#### Tail Pinch Reflex

Beginning on PND30, each pup was placed on a board and received a tail pinch with a hemostatic forceps (wrapped with foam) with a pinching force for up to 20 s. The pinching force applied was not too strong as to hurt the pups. The first day when animals began to bite or lick the hemostatic forceps was recorded.

### Autistic Behavioral Testing

#### Social Interaction Test

PND 45 pups were introduced to a 3-chamber social interaction apparatus. Openings between the compartments allowed the animals access all three chambers. In the first phase, a pup was placed in the 3-chambered apparatus and allowed to explore the environment freely for 10 min for habituation. After the habituation phase, the subject was gently guided to the central chamber, and the two entrances were blocked. Two small containers with either an object (Object) or an age-matched rat (Stranger #1) were placed in the left and right chambers. Then, the two entrances were opened to allow the rat in the center to explore the new environment freely for 10 min. In the third phase, the test rat was gently guided to the center chamber again, and the entrances were blocked. The Object was replaced with an age-matched rat (Stranger #2), and the test rat was then allowed to explore Stranger #1 and #2 for additional 10 min. Time spent in each chamber was measured using homemade software. Individual movement tracks were recorded by using a video system and analyzed using homemade software. The Sociability Index (SI) was defined as the ratio between the time spent in the chamber with the stranger rat and time spent in the chamber with the Object during the second phase of the test. The apparatus was cleaned with 70% ethanol and water in between trials.

#### Open-Field Test

Open field test was conducted at PND40. Pups were accommodated for 1 h before testing in a quiet room under adjusted lighting. A square wooden box (100 cm × 100 cm × 40 cm) was used for this locomotor activity test. The floor area was divided into 25 blocks of equal size with 9 blocks making up the center grid. The pups were allowed to accommodate in the box for 5 min and then placed inside the central block and its movements monitored with a video camera for 10 min. The number of blocks that pup passed through (cross grid) and the frequency of straight upward movements (vertical) were recorded. Defecation and urination frequency of animals were also measured. The open field was thoroughly cleaned with 70% alcohol between test animals.

#### Self-Grooming

Self grooming was conducted at PND35, and each pups was placed individually into a clean square wooden box (100 cm × 100 cm × 40 cm) with a video camera placed 15 cm away from the cage. The behavior of the rats was recorded for 10 min after accommodation to the test room for 1 h and being housed in the box for 5 min before testing. The time pups spent grooming was recorded.

#### Morris Water Maze

Morris water maze was performed from PND35 to PND41, including platform navigation and spatial probe tests to detect the spatial learning and memory ability in the rats. The water maze was a circular pool (120 cm in diameter equipped with a 9 cm diameter platform) and filled with water at 25 ± 1°C. The test consisted of the acquisition phase during the first 6 days and a probe trial on the seventh day.

#### Place Navigation Test

This test was carried out for 6 days, and the time per trial was 60 s. The pups had four trials in each day to find the platform, after placement in the water in one of four quadrants. The pup was allowed to look for the platform for 60 s. If it managed to find the platform, it was allowed to remain on the platform for 20 s. If it could not find the platform within 60 s, the animal would be guided by the experimenter to the platform and allowed to stay on the platform for 20 s. The time taken to find the platform was considered to be the escape latency, as measured by using the SMART video tracking system (SMART v3.0, Panlab, Spain).

#### Spatial Probe Test

On the seventh days of testing, a spatial probe test was performed in which the platform was removed. The pups had four trials to find the platform after placement in the water in one of four quadrants and being allowed to swim freely for 60 s. The number of crossings of the former platform location (passing time), the time spent in the target quadrant (time ratio in target quadrant) and swimming speed were measured by using the SMART video tracking system (SMART v3.0, Panlab, Spain).

### Western Blot

Western blotting was used to detect the expression of GAD67 in different encephalic regions of collected rat brains. Total protein lysates made from different encephalic regions were mixed with SDS gel-loading buffer and heated for 5 min at 100°C. Samples (50 μg protein in each group) were separated on 8% SDS-PAGE gels (Invitrogen, Carlsbad, CA, USA), and transferred to polyvinylidene difluoride membranes (Millipore, Bedford, MA, USA). The membranes were blocked for 1 h at room temperature with 5% nonfat milk in TBST (TBS containing 0.05% Tween 20), and then probed with specific primary antibody (anti-GAD67, 1:1,000, CST, USA) for overnight at 4°C. A horseradish peroxidase (HRP)-conjugated secondary antibody was then added for 2 h at room temperature. Immuno-positive GAD67 bands were scanned and densitometrically analyzed by automated ImageJ software (NIH Image, Version 1.61), and their total protein densities were expressed relative to β-actin signals. GAD67 content for each brain region was measured five times.

### Immunohistochemistry

To study the GAD67 protein in the brain, we performed IHC and DAB staining to demonstrate the presence and location of GAD67 proteins in different encephalic regions. The brain was harvested and processed by formalin fixation and paraffin-embedding, and 4 μm sections were cut for IHC. Sections were deparaffinized and rehydrated with xylene and ethyl alcohol, and then subjected to H_2_O_2_ treatment of endogenous peroxidase, high pressure antigen retrieval, and protein blocking. Sections were incubated with specific primary antibody (anti-GAD67, 1:1,000, CST, USA) for overnight at 4°C, and then incubated with the secondary antibody (biotinylated goat anti-rabbit IgG, 1:100) for 60 min at room temperature. Next, sections were incubated with streptavidin-HRP (S-A/HRP) followed by DAB chromogen and Mayer’s hematoxylin. Images were collected under a microscope and the mean densities of GAD67 immunostaining were measured by using Image Pro Plus software.

### Statistical Analysis

All data were presented as mean ± SEM. Statistical analyses were carried out using Graphpad Prism 7.0 software. Significant differences between groups were determined using Student’s *t*-test and Repeated measures Two-way ANOVA followed by LSD post-test or Bonferroni post-test. Values of *p* < 0.05 were regarded as the criterion of significance.

## Results

### VPA-Treated Rats Exhibited Delayed Nervous Reflex Development

In order to observe the neurotoxic effect of VPA on the VPA-treated offspring rats, the nervous reflexes were tested. As shown in Figure 1B, VPA-treated rats showed normal performance in the first pivoting appearance day compared with control rats (2.75 ± 0.30 days in control rats (*n* = 2 mothers, 12 pups) vs. 2.91 ± 0.19 days in VPA rats (*n* = 3 mothers, 24 pups), *t*-test, *t* = 0.4708, *df* = 34, *p* = 0.6408). VPA-treated rats exhibited significantly longer latencies in behavioral ontogeny than control rats, including the first appearance day of surface righting reflex (3.08 ± 0.08 days in control rats (*n* = 2 mothers, 12 pups) vs. 3.58 ± 0.15 days in VPA rats (*n* = 3 mothers, 24 pups), *t*-test, *t* = 2.147, *df* = 34, *p* = 0.039; Figure 1C), negative geotaxis reflex (4.33 ± 0.14 days in control rats (*n* = 2 mothers, 12 pups) vs. 5.75 ± 0.22 days in VPA rats (*n* = 3 mothers, 24 pups), *t*-test, *t* = 4.185, *df* = 34, *p* = 0.00019; Figure 1D), cliff aversion (5.08 ± 0.08 days in control rats (*n* = 3 mothers, 12 pups) vs. 5.79 ± 0.21 days in VPA rats (*n* = 3 mothers, 24 pups), *t*-test, *t* = 2.253, *df* = 34, *p* = 0.03079; Figure 1E) and air righting reflex (7.41 ± 0.19 days in control rats (*n* = 3 mothers, 12 pups) vs. 8.75 ± 0.17 in VPA rats (*n* = 3 mothers, 24 pups), *t*-test, *t* = 4.752, *df* = 34, *p* < 0.0001; Figure 1F). These results indicate that prenatally treated VPA causes a neurotoxic effect to impair rat offspring nervous reflex development.

### VPA-Treated Rats Showed Altered Motor Coordination and Delayed Sensory Development

**Figure 2:**
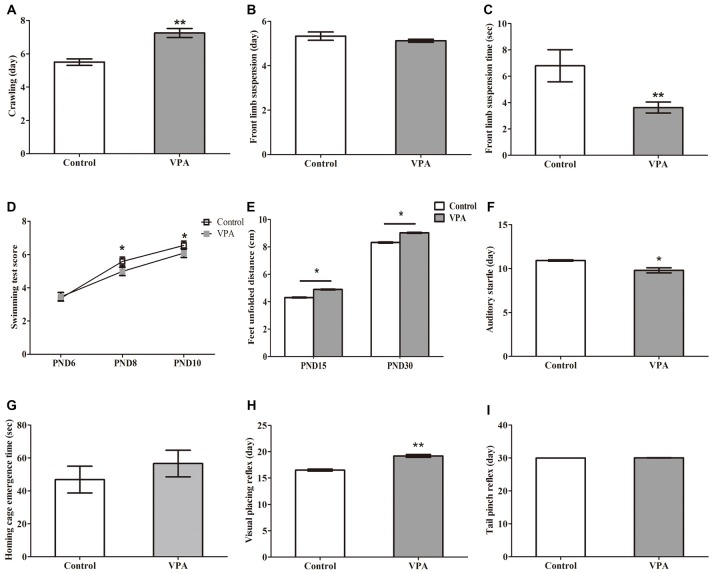
VPA-treated rats showed altered motor coordination and delayed sensory development. **(A)** VPA-treated rats displayed significant delayed crawling in the first appearance day (**p < 0.01). **(B)** No difference was observed in the first appearance day of front limb suspension in both control and VPA-treated rats (*p* > 0.05). **(C)** The time of front limb suspension is significant shorter in VPA-treated group than control group (**p < 0.01). **(D)** VPA-treated rats show significant lower swimming scores at postnatal day 8 (PND8; *p < 0.05) and PND10 (*p < 0.05) compared to control matched rats, respectively, but no difference is observed at PND6 between control rats and VPA-treated rats (*p* > 0.05). **(E)** The distance of feet unfolded is significant longer at PND15 (**p < 0.01) and PND30 (**p < 0.01) in VPA-treated rats than that of control matched rats, respectively. **(F)** The first time of the auditory startle appeared to occur earlier in the VPA-treated rats than in control rats (*p < 0.05). **(G)** No difference was measured in time of home cage emergence between two groups (*p* > 0.05). **(H)** Compared with the control group, the appearance day of visual placing reflex were significant later in the VPA group (**p < 0.01). **(I)** The tail pinch reflex appeared to occur in same time of development in both two groups (*p* > 0.05).

We next examined the influence of prenatal treatment with VPA on offspring animal motor coordination and sensory development. As illustrated in Figures 2A, VPA-treated rats displayed significant delayed crawling in the first appearance day (5.50 ± 0.19 days in control rats (*n* = 2 mothers, 12 pups) vs. 7.25 ± 0.27 days in VPA rats (*n* = 3 mothers, 24 pups), *t*-test, *t* = 4.279, *df* = 34, *p* = 0.00014). Interestingly, no difference was observed in the first appearance day of front limb suspension in both control and VPA-treated rats (5.33 ± 0.19 days in control rats (*n* = 2 mothers, 12 pups) vs. 5.12 ± 0.07 days in VPA rats (*n* = 3 mothers, 24 pups), *t*-test, *t* = 1.272, *df* = 34, *p* = 0.2119; Figure 2B). The time of front limb suspension was significantly shorter in VPA-treated group than in control group (6.79 ± 1.22 s in control rats (*n* = 2 mothers, 12 pups) vs. 3.62 ± 0.41 s in VPA rats (*n* = 3 mothers, pups 24), *t-test*, *t* = 3.06, *df* = 34, *p* = 0.0043; Figure 2C). VPA-treated rats showed significantly lower swimming scores at the PND8 (5.66 ± 0.14 in control rats (*n* = 2 mothers, 12 pups) vs. 5.29 ± 0.09 in VPA rats (*n* = 3 mothers, 24 pups), *t*-test, *t* = 2.24, *df* = 34, *p* = 0.031) and PND10 (6.25 ± 0.13 in control rats (*n* = 2 mothers, 12 pups) vs. 5.79 ± 0.12 in VPA rats (*n* = 3 mothers, 24 pups), *t*-test, *t* = 2.366, *df* = 34, *p* = 0.023) compared to control matched rats, respectively, but no difference was observed at the PND6 between control rats and VPA-treated rats (3.16 ± 0.11 in control rats vs. 3.45 ± 0.10 in VPA rats, *t*-test, *t* = 1.742, *df* = 34, *p* = 0.090; Figure 2D). As shown in Figure 2E, feet unfolded distance was significantly longer at the PND15 (4.31 ± 0.09 cm in control rats (*n* = 2 mothers, 12 pups) vs. 4.89 ± 0.12 cm in VPA rats (*n* = 3 mothers, 24 pups), *t*-test, *t* = 3.052, *df* = 34, *p* = 0.004) and PND30 (8.31 ± 0.13 cm in control rats (*n* = 2 mothers, 12 pups) vs. 9.02 ± 0.15 cm in VPA rats (*n* = 3 mothers, 24 pups), *t*-test, *t* = 3.036, *df* = 34, *p* = 0.004) in VPA-treated rats than that of control matched rats, respectively. The first time of the auditory startle appeared to occur earlier in the VPA-treated rats than in control rats (10.91 ± 0.08 days in control rats (*n* = 2 mothers, 12 pups) vs. 9.79 ± 0.29 days in VPA rats (*n* = 3 mothers, 24 pups), *t*-test, *t* = 2.654, *df* = 34, *p* = 0.012; Figure 2F). However, no difference was measured in time of home cage emergence between two groups (46.83 ± 8.12 s in control rats (*n* = 2 mothers, 12 pups) vs. 56.58 ± 8.07 s in VPA rats (*n* = 3 mothers, 24 pups), *t*-test, *t* = 0.7603, *df* = 34, *p* = 0.452; Figure 2G). Compared with the control group, the appearance day of visual placing reflex was significantly delayed in the VPA group (16.50 ± 0.19 days in control rats (*n* = 2 mothers, 12 pups) vs. 18.37 ± 0.41 days in VPA rats (*n* = 3 mothers, 24 pups), *t*-test, *t* = 3.114, *df* = 34, *p* = 0.003; Figure 2H). The tail pinch reflex appeared to occur at the same time of development in both two groups (30.00 ± 0.00 days in control rats (*n* = 2 mothers, 12 pups) vs. 30.04 ± 0.04 days in VPA rats (*n* = 3 mothers, 24 pups), *t*-test, *t* = 0.702, *df* = 34, *p* = 0.487; Figure 2I). These findings demonstrate that VPA exposure during prenatal stage leads to impairments in both motor coordination and sensory development in the offspring animals.

### VPA-Treated Rats Exhibited Autistic-Like Behaviors

**Figure 3:**
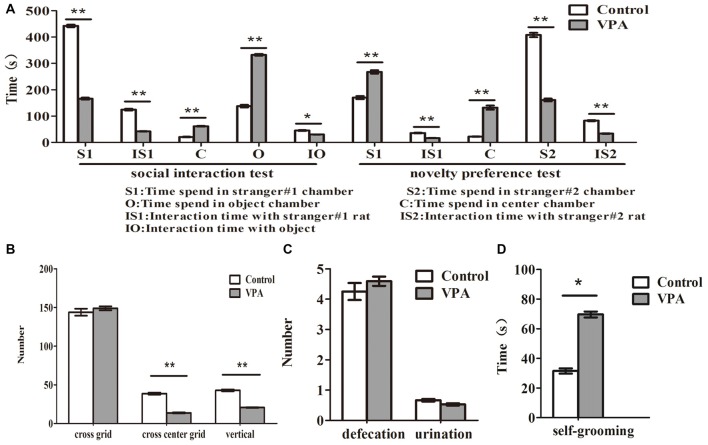
VPA-treated rats exhibited autistic-like behaviors. **(A)** Compared with the control rats, VPA-treated rats spent less time to stay in stranger#1 chamber (**p < 0.01) and to interact with stranger#1 rats (**p < 0.01), and more time to interact with object (*p < 0.05) and center chamber (**p < 0.01) in 3-chamber social interaction test, respectively. In novelty preference test, VPA-treated rats spent more time to stay in strange#1 chamber (**p < 0.01) and center chamber (**p < 0.01) and less time to interact with strange#1 rats (**p < 0.01), respectively. Meanwhile, VPA-treated rats had significantly impaired preference to stay in stranger#2 chamber (**p < 0.01) and spent less time to interact with stranger#2 rats (**p < 0.01), in 3-chamber social preference test, respectively. **(B)** VPA-treated rats exhibited anxiety-like behaviors in the open field test, indicating that VPA rats have abnormal exploratory behaviors for novelty—less numbers in the cross-center grid (**p < 0.01) and vertical grid (**p < 0.01), but no changed in the cross-total grid number (*p* > 0.05), compared with matched control rats. **(C)** No differences were observed in the numbers of defecation (*p* > 0.05) and urination (*p* > 0.05) between VPA and control rats in the open-field test. **(D)** VPA-treated rats exhibited a more grooming behaviors than control rats in the self-grooming test (*p < 0.05).

Compared with the control rats, VPA-treated rats spent less time to stay in stranger#1 chamber (442.33 ± 16.45 s in control rats (*n* = 2 mothers, 12 pups) vs. 166.06 ± 14.73 s in VPA rats (*n* = 3 mothers, 16 pups), *t*-test, *t* = 12.45, *df* = 26, *p* < 0.0001) and to interact with stranger #1 rats (124.41 ± 11.34 s in control rats (*n* = 2 mothers, 12 pups) vs. 41.93 ± 3.01 s in VPA-treated rats (*n* = 3 mothers, 16 pups), *t*-test, *t* = 7.959, *df* = 26, *p* < 0.0001), and more time to interact with object (137.6 ± 17.36 s in control rats (*n* = 2 mothers, 12 pups) vs. 332.5 ± 13.44 s in VPA-treated rats (*n* = 3 mothers, 16 pups), *t*-test, *t* = 9.028, *df* = 26, *p* < 0.0001) and center chamber (20.08 ± 4.49 s in control rats (*n* = 2 mothers, 12 pups) vs. 61.43 ± 6.11 s in VPA-treated rats (*n* = 3 mothers, 24 pups), *t*-test, *t* = 5.119, *df* = 26, *p* < 0.0001) in 3-chamber social interaction test, respectively (Figure 3A). In novelty preference test, VPA-treated rats spent more time to stay in strange#1 chamber (170.08 ± 20.59 s in control rats (*n* = 2 mothers, 12 pups) vs. 267.50 ± 25.71 s in VPA rats (*n* = 3 mothers, 16 pups), *t*-test, *t* = 2.808, *df* = 26, *p* = 0.009) and center chamber (22.00 ± 3.19 s in control rats (*n* = 2 mothers, 12 pups) vs. 132.06 ± 32.17 s in VPA rats (*n* = 3 mothers, 24 pups), *t*-test, *t* = 2.941, *df* = 26, *p* = 0.006) and less time to interact with strange #1 rats (35.75 ± 3.89 s in control rats (*n* = 2 mothers, 12 pups) vs. 16.43 ± 2.02 s in VPA rats (*n* = 3 mothers, 24 pups), *t*-test, *t* = 4.717, *df* = 26, *p* < 0.0001), respectively (Figure 3A). Meanwhile, VPA-treated rats had significantly impaired preference to stay in stranger#2 chamber (407.91 ± 28.73 s in control rats (*n* = 2 mothers, 12 pups) vs. 161.00 ± 21.72 s in VPA rats (*n* = 3 mothers, 16 pups), *t*-test, *t* = 6.995, *df* = 26, *p* < 0.0001) and spent less time to interact with stranger #2 rats (82.75 ± 6.95 s in control rats (*n* = 2 mothers, 12 pups) vs. 33.43 ± 4.86 s in VPA rats (*n* = 3 mothers, 16 pups), *t*-test, *t* = 5.997, *df* = 26, *p* < 0.0001), in 3-chamber social preference test, respectively (Figure 3A). VPA-treated rats exhibited anxiety-like behaviors in the open field test, indicating that VPA rats had abnormal exploratory behaviors for novelty—less numbers in the cross center grid (38.67 ± 4.45 times in control rats (*n* = 2 mothers, 12 pups) vs. 13.70 ± 2.97 times in VPA rats (*n* = 3 mothers, 17 pups, *t*-test, *t* = 5.029, *df* = 27, *p* < 0.0001) and vertical grid (42.91 ± 3.94 times in control rats (*n* = 2 mothers, 12 pups) vs. 20.64 ± 1.68 times in VPA rats (*n* = 3 mothers, 17 pups), *t*-test, *t* = 5.772, *df* = 27, *p* < 0.0001), but no changed in the cross total grid number (143.83 ± 15.65 times in control rats (*n* = 2 mothers, 12 pups) vs. 148.88 ± 10.61 times in VPA rats (*n* = 3 mothers, 17 pups), *t*-test, *t* = 0.2774, *df* = 27, *p* = 0.783), compared with matched control rats (Figure 3B). As shown in Figure 3C, no differences were observed in the numbers of defecation (4.25 ± 0.97 times in control rats (*n* = 2 mothers, 12 pups) vs. 4.58 ± 0.63 times in VPA rats (*n* = 3 mothers, 17 pups), *t*-test, *t* = 0.3058, *df* = 27, *p* = 0.762) and urination (0.66 ± 0.14 ml in control rats (*n* = 2 mothers, 12 pups) vs. 0.52 ± 0.15 ml in VPA rats (*n* = 3 mothers, 17 pups), *t*-test, *t* = 0.634, *df* = 27, *p* = 0.531) between VPA and control rats in the open-field test. VPA-treated rats also exhibited a more grooming behaviors than control rats in the self-grooming test (31.50 ± 6.12 s in control rats (*n* = 2 mothers, 12 pups) vs. 69.58 ± 9.94 s in VPA rats (*n* = 3 mothers, 24 pups), *t*-test, *t* = 2.573, *df* = 34, *p* = 0.014; Figure 3D). These data suggest that prenatally treated VPA rats show behavioral deficits in the offspring similar with core autistic behaviors in the patients with autism (American-Psychiatric-Association, 2013).

### VPA Rats Exhibited Spatial Learning and Memory Impairment in Morris Water Maze Test

**Figure 4:**
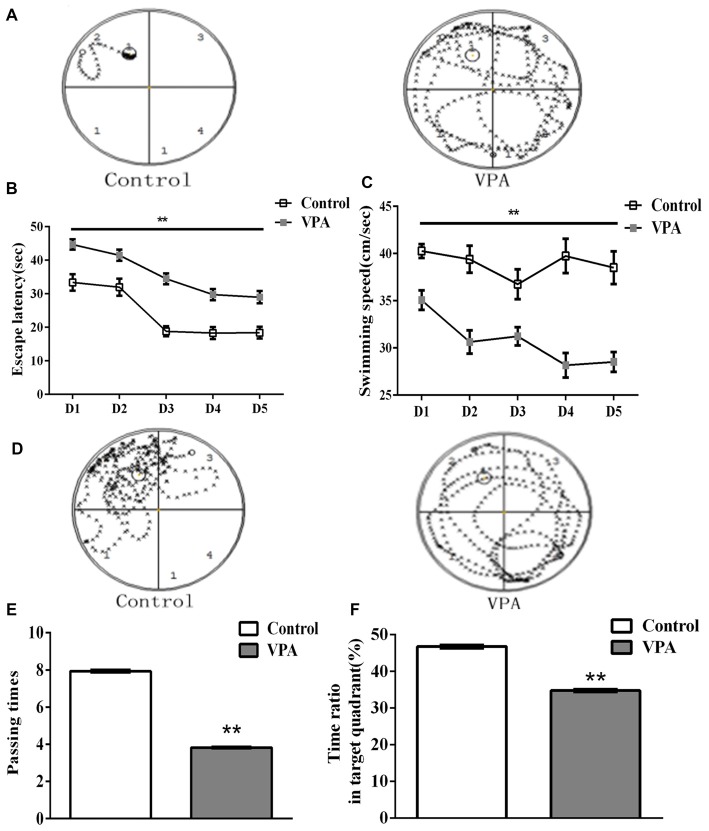
VPA rats exhibited spatial learning and memory impairment in Morris water maze test. ** (A)** Representative swimming paths of control (left) and VPA-treated (right) rats during the probe trial session in the Morris water maze test. **(B)** VPA-treated rats had significantly longer escape latency to find the platform than that in control rats (**p < 0.01). **(C)** VPA-treated rats exhibited slower swimming speed than control rats in the place navigation test (**p < 0.01). **(D)** Swimming path map of control (left) and VPA-treated (right) crossing different quadrants after removing the platform in the Morris water maze test. **(E)** VPA rats displayed a reduced number of platform passing (**p < 0.01). **(F)** VPA-treated rats exhibited less time in target quadrant compared with control rats (**p < 0.01).

HC-dependent spatial learning and memory was evaluated by the Morris water maze test in VPA-treated rat offspring. Figure 4A showed the representative swimming paths of control (left) and VPA-treated (right) rats during the probe trial session in the Morris water maze test. Since the same rats had been used in this test, we applied a repeated measures two-way ANOVA to analyze animal spatial learning and memory. As shown in Figure 4B, VPA-treated rats had significantly different escape latency to find the platform than that in control rats (repeated measures two-way ANOVA, *F*_(1,33)_ = 45.43, **p < 0.0001). We also analyzed the interaction between time (days) and treatment (saline and VPA) on the total variance in Figure 4B, showing that there is no significant interaction between time (days) and treatment (saline- and VPA-treated; Interaction, *F*_(4,132)_ = 1.141, *p* = 0.3401). Further detailed information showed that interaction accounted for 0.81% of the total variance and was considered not significant, indicating that time has the same effect on all animals including control and VPA-treated rats. The data also showed that VPA treated accounted for 25.44% of the total variances (after adjusting for matching). Taken together, these data variance should be accounted from VPA treatment, not from different time measurement. The VPA effect is considered extremely significant in Figure 4B, suggesting that VPA-treated rats had significantly longer escaped latency to find the platform than that in control rats. VPA-treated rats also exhibited slower swimming speed than control rats in the place navigation test (repeated measures two-way ANOVA, *F*_(1,33)_ = 60.52, **p < 0.0001; Figure 4C). Figure 4D showed the representative maps of rats crossing different quadrants after removing the platform in the Morris water maze test. As shown in Figure 4E, VPA rats displayed a reduced number of platform passing (7.93 ± 0.07 times in control rats (*n* = 2 mothers, 15 pups) vs. 3.82 ± 0.03 times in VPA rats (*n* = 3 mothers, 20 pups), *t*-test, *t* = 59.03, *df* = 33, *p* < 0.0001). VPA-treated rats also exhibited less time in target quadrant compared with control rats (46.74 ± 0.45% in control rats (*n* = 2 mothers, 15 pups) vs. 34.73 ± 0.32% in VPA rats (*n* = 3 mothers, 20 pups), *t*-test, *t* = 22.38, *df* = 33, *p* < 0.0001; Figure 4F). These findings reveal that HC-dependent spatial learning and memory are impaired in this animal model of autism.

### Altered Expression of GAD67 Measured by Western Blotting in Different Brain Regions in the Development of the Central Nervous System

**Figure 5:**
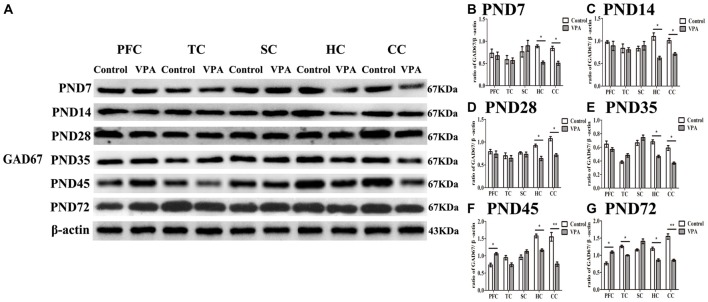
Altered expression of glutamic acid decarboxylase 67 (GAD67) measured by western blotting in different brain regions in the development of the central nervous system. **(A)** Representative WBs were shown to measure GAD67 protein expression in the prefrontal cortex (PFC), temporal cortex (TC), somatosensory cortex (SC), hippocampus (HC) and cerebellum cortex (CC) in both control and VPA-treated rats at the PND7, PND14, PND35, PND45 and PND72, respectively. **(B–G)** Quantification of GAD67 expression in PFC indicated that no changes occurred at the early stage of development including PND7 (*p* > 0.05), PND14 (*p* > 0.05), PND28 (*p* > 0.05) and PND35 (*p* > 0.05), in VPA-treated rats. Interestingly, VPA-treated rats had the increased GAD67 expression at the young adult stage such as PND45 (*p < 0.05) and PN72d (*p < 0.05) compared with matched control rats. GAD67 expression in TC in two groups was same at the PND7, PND14, PND28, PND35 and PND45, but there was low expression of GAD67 in TC in VPA group than that in control group at the PND72 (*p < 0.05). There were no significant differences of the GAD67 expression in SC in two groups at all the test time points. Notably, GAD67 expressions in both HC (*t*-test, *p < 0.05) and CC (*t*-test, *p < 0.05 and **p < 0.01) were lower in VPA group than in control group at all the test time points.

To investigate the relationship between the delayed neural development accompanied with altered behaviors and changes in the GABAergic system in the brain of rats prenatally affected by VPA, we measured GAD67 expression using western blotting in different brain regions at the different development stages. In Figure 5A, representative WBs were shown to measure GAD67 protein expression in the PFC, TC, somatosensory cortex (SC), HC and cerebellum cortex (CC) in both control and VPA-treated rats at the PND7, PND14, PND35, PND45 and PND72, respectively. As shown in Figures 5B–G, quantification of GAD67 expression in PFC indicated that no changes occurred at the early stage of development including PND7 (0.73 ± 0.17 in control rats (*n* = 2 mother, 3 pups) vs. 0.67 ± 0.14 in VPA rats (*n* = 3 mothers, 3 pups), *t*-test, *t* = 0.2507, *df* = 4, *p* = 0.814), PND14 (0.97 ± 0.05 in control rats (*n* = 2 mother, 3 pups) vs. 0.89 ± 0.18 in VPA rats (*n* = 3 mothers, 3 pups), *t*-test, *t* = 0.4556, *df* = 4, *p* = 0.672), PND28 (0.78 ± 0.08 in control rats (*n* = 3 mothers, 3 pups) vs. 0.73 ± 0.11 in VPA rats (*n* = 3 mothers, 3 pups), *t*-test, *t* = 0.3655, *df* = 4, *p* = 0.733) and PND35 (0.64 ± 0.08 in control rats (*n* = 3 mothers, 3 pups) vs. 0.56 ± 0.04 in VPA rats (*n* = 3 mothers, 3 pups), *t*-test, *t* = 0.8556, *df* = 4, *p* = 0.440), in VPA-treated rats. Interestingly, VPA-treated rats had the increased GAD67 expression in the PFC at the young adult stage such as PND45 (0.73 ± 0.09 in control rats (*n* = 2 mothers, 3 pups) vs. 1.06 ± 0.06 in VPA rats (*n* = 3 mothers, 3 pups), *t*-test, *t* = 3.022, *df* = 4, *p* = 0.039) and PND72 (0.76 ± 0.07 in control rats (*n* = 3 mothers, 3 pups) vs.1.09 ± 0.06 in VPA rats (*n* = 3 mothers, 3 pups), *t*-test, *t* = 3.44, *df* = 4, *p* = 0.026) compared with matched control rats. GAD67 expression in TC in two groups was same at PND7, PND14, PND28, PND35 and PND45, but there was low expression of GAD67 in TC in VPA group than that in control group at PND72 (1.25 ± 0.06 in control rats (*n* = 3 mothers, 3 pups) vs. 0.99 ± 0.03 in VPA rats (*n* = 3 mothers, 3 pups), *t*-test, *t* = 3.929, *df* = 4, *p* = 0.0171). There were no significant differences of the GAD67 expression in SC in two groups at all the test time points. Notably, GAD67 expressions in both HC (*p* < 0.05) and CC (*p* < 0.05 or *p* < 0.01) were lower in VPA group than in control group at all the test time points. These results indicate that early embryonic exposure to VPA in rats leads to dysregulate the GABAergic system in the brain of offspring rats, especially in the HC and cerebellum all the time, and in the PFC in early adulthood, but not at an early development stage.

### VPA Treatment Resulted in Altered Brain GAD67 Expression Measured by Immunohistochemistry

**Figure 6:**
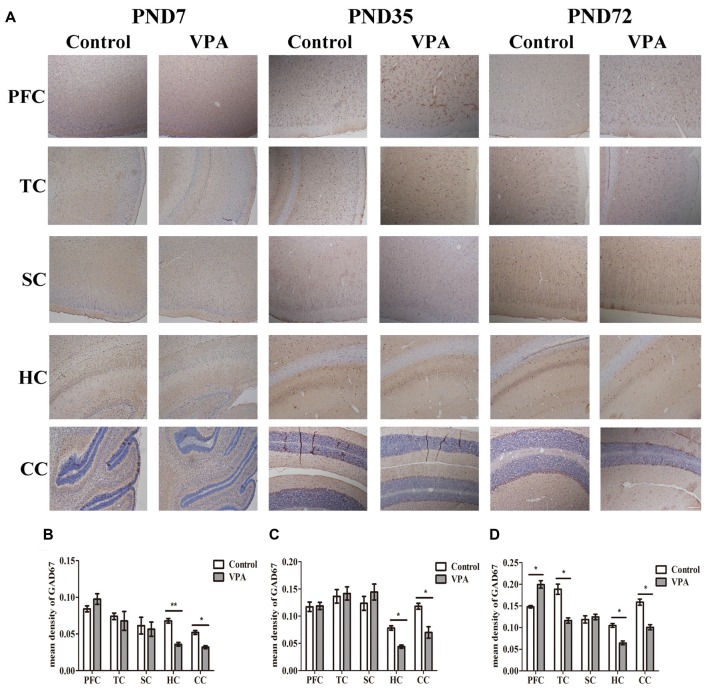
VPA treatment resulted in altered brain GAD67 expression measured by IHC. **(A)** Representative images of GAD67 expression is measured by IHC at PND7, PND35 and PND72 in the different brain regions of both control group and VPA group. **(B–D)** IHC staining of brain sections indicated that VPA rats exhibited significantly lower expression of GAD67 in HC and CC at above three time points compared to control rats (*p < 0.05 or **p < 0.01). No differences were observed in GAD67 expression in the PFC and TC of both control and VPA-treated rats at PND7 and PND35. Interestingly, higher GAD67 expression was measured in the PFC of the VPA rats at PND72 (*p < 0.05) while lower GAD67 expression was detected in the TC of the VPA rats at the same time (*p < 0.05). No changes of GAD67 expression were observed in SC between control and VPA rats at these three time points. Scale bar = 300 μm.

We further employed IHC to define changes in the GABAergic system of VPA rats’ brain. Figure 6A showed the representative images of GAD67 expression measured by IHC at PND7, PND35 and PND72 in the different brain regions of both control group and VPA group. As illustrated in Figures 6B–D, VPA rats exhibited significantly lower expression of GAD67 in HC and CC at above three time points compared to control rats (0.067 ± 0.006 in HC; 0.052 ± 0.006 in CC in control rats (*n* = 3 mothers, 5 pups) vs. 0.035 ± 0.005 in HC, 0.032 ± 0.004 in CC in VPA rats (*n* = 3 mothers, 5 pups), *t*-test, *t* = 3.609, *df* = 8, *p* = 0.0068 and *t* = 2.724, *df* = 8, *p* = 0.0260, respectively, at PND7; 0.078 ± 0.008 in HC, 0.118 ± 0.012 in CC in control rats (*n* = 3 mothers, 5 pups) vs. 0.044 ± 0.006 in HC, 0.070 ± 0.011 in CC in VPA rats (*n* = 3 mothers, 5 pups), *t*-test, *t* = 3.046, *df* = 8, *p* = 0.0159 and *t* = 2.852, *df* = 8, *p* = 0.0214, respectively, at PND35; and 0.105 ± 0.009 in HC, 0.158 ± 0.016 in CC in control rats (*n* = 3 mothers, 5 pups) vs. 0.064 ± 0.009 in HC, 0.100 ± 0.013 in CC in VPA rats (*n* = 3 mothers, 5 pups), *t*-test, *t* = 2.974, *df* = 8, *p* = 0.0177 and *t* = 2.812, *df* = 8, *p* = 0.0227, respectively, at PND72). No differences were observed in GAD67 expression in the PFC and TC of both control and VPA-treated rats at PND7 and PND35. Interestingly, higher GAD67 expression was measured in the PFC of the VPA rats (0.140 ± 0.006 in control rats (*n* = 3 mothers, 5 pups) vs. 0.199 ± 0.019 in VPA rats (*n* = 3 mothers, 5 pups), *t*-test, *t* = 2.517, *df* = 8, *p* = 0.0359) while lower GAD67 expression was detected in the TC of the VPA rats at PND72 (0.188 ± 0.026 in control rats (*n* = 3 mothers, 5 pups) vs. 0.116 ± 0.014 in VPA rats (*n* = 3 mothers, 5 pups), *t*-test, *t* = 2.415, *df* = 8, *p* = 0.0421). No changes of GAD67 expression were observed in SC between control and VPA rats at all three tested time points. This study provides further evidence to support the western blotting results that the GABAergic systems in the brain, particularly in the HC, cerebellum and PFC, are dysregulated in this animal model of autism.

## Discussion

The goal of the present study was to investigate the role of GABAergic signaling in the neural development of rats and their behaviors relevant to autism. In the present study, we used a VPA-treatment induced rat model of autism to evaluate autism-like behaviors and GABAergic signal underlying these altered behaviors in multiple brain areas at the developmental stages from birth to adulthood. Our findings confirm that VPA exposure *in utero* results in behavioral deficits relevant to autism, including delayed nervous reflex development, motor coordination deficiencies, delayed sensory development, anxiety-like behavior, less exploratory behavior and diminished social preference, elevated self-grooming and impaired spatial learning and memory. We also present evidence that prenatal VPA exposure dysregulates expression of GAD67, a GABA producing enzyme, in the cerebral cortex, HC and cerebellum. Our findings indicate that these discrete behavioral abnormalities correlate with reduced GABAergic signaling that is associated with abnormal excitatory-inhibitory balance, which could impact developmental synaptic plasticity and neural circuit formation in multiple brain regions. This study also provides a strong indication that enhancing GABA signaling could be a clinically relevant pharmacological rescue strategy for treatment of individuals with autism.

A considerable amount of clinical evidence suggests that autism is also accompanied by many forms of developmental delays, including impaired gross and fine motor skills (Provost et al., 2007), postponed sensorimotor formation (Moog et al., 2004), deficits in joint attention (Bernabei et al., 2003), and altered adaptive behavior (Fenton et al., 2003). VPA is both an anti-convulsant and a mood stabilizer; however, these pharmacological properties may not have the same underlying biochemical mechanism (Rapoport, 2014). Clinical studies have shown that exposure to VPA *in utero* is associated with birth defects, cognitive deficits, and increased risk of autism (Nadebaum et al., 2011). Our data demonstrate that VPA-treated rats also exhibited autism-like behaviors and deficits in the reflex development, motor coordination, auditory and visual development, which are consistent with clinical symptoms of patients with autism and the findings of other labs’ using VPA-induced rodent models of autism (Snow et al., 2008; Mehta et al., 2011; Favre et al., 2013; Olexová et al., 2013, 2016; Ha et al., 2017).

Although the exact mechanisms of anxiety disorders are still only partially understood, many studies have shown that an increase in anxiety is accompanied by GABAergic system deficits (Lydiard, 2003; Gafford et al., 2012). Anxiety is one of the characteristic symptoms of autism (Lang et al., 2010; Vasa et al., 2014), which could be attributed to a dysregulated GABAergic system in the brain (Olexová et al., 2016). Our research showed that adult VPA rats spent less time in the inner zone of the test chamber during the open field test, which is an indication of anxiety-like behaviors in the animals. By using western blotting and histochemical analyses, we also found that VPA rats at ages from PND7 to PND72 (from childhood to adulthood) had decreased expression of GAD67 in the HC and cerebellum; whereas, decreased GAD67 expression was only observed in the TC at PND72. Conversely, GAD67 expression was increased in the PFC at PND45 and PND72. Numerous studies have demonstrated that the medial PFC (mPFC) and the ventral HC (vHPC), but not the dorsal HC (dHPC), play a key role in brain processes associated with anxiety-related behavior (Kjelstrup et al., 2002; Bannerman et al., 2004; Adhikari et al., 2010). The mPFC receives direct projections from the vHPC in both rats (Jay et al., 1989; Verwer et al., 1997) and mice (Parent et al., 2010); where the vHPC-mPFC connection is important for the modulation of anxiety-related behaviors (Adhikari et al., 2010). Our study showed that decreased inhibitory GABA signaling in the HC could result in an excitation-inhibition imbalance, causing abnormal synaptic plasticity and neural network formation that would disturb hippocampal function. This further supports the proposition that altered hippocampal activity is associated with autism-related behavior. Our research also revealed that there are no obvious changes in the GAD67 expression of the mPFC in early-stage development (from PND7 to PND35); whereas, there is a marked alteration of the GAD67 expression of the mPFC in the adolescence (PND45) and adulthood (PND72) phases. Since the reduced expression of GAD67 occurred in early development of the HC, one possibility is that the vHPC sends excessive information flow to the mPFC, which interferes with its development; and thus, leads to an upregulation of GABAergic signaling in the mPFC in an attempt to restore the excitation-inhibition imbalance of the hippocampal-mPFC connection during adolescence and adulthood. However, this could further lead to a hypofunction of the mPFC, which in turn aggravates anxiety-related behaviors in the VPA-treated offspring rats. Overall, our findings strongly support the existence of a relationship between increased anxiety-like behavior and changes in the regulation of the GABAergic system in VPA rats.

A subpopulation of patients afflicted with ASD, who are defined as high functioning individuals, display superior abilities in very specific and focused cognitive functions; however, the majority of individuals with autism exhibit global cognitive delays and significant language impairments (Gray and Tonge, 2005; Desombre et al., 2006; Baranek et al., 2007; Pilowsky et al., 2007; Ellis Weismer et al., 2010) as well as self-recognition deficits (Ferrari and Matthews, 1983). It is widely accepted that the dorsal HC has a privileged role in certain forms of learning and memory, particularly spatial learning, but the ventral HC plays a preferential role in brain processes associated with anxiety-related behaviors (Bannerman et al., 2004; Parent et al., 2010; Kempadoo et al., 2016). A recent study has shown that direct vHPC-mPFC afferent pathways are critical for spatial encoding in working memory (Spellman et al., 2015), supporting the premise that a developmental lesion of the ventral HC impairs performance in spatial working memory related to the PFC (Lipska et al., 2002). Autistic individuals often maintain rigid habits and frequently show a strong insistence on sameness and become distraught by a change in routine (Crawley, 2007). Individuals with ASD have impaired navigation skills that are a result of difficulties generating cognitive maps of their environment (Lind et al., 2013). Our studies, using the Morris Water Maze, showed that VPA rats not only have delayed memory acquisition skills, but are also resistant to sudden changes in memory tasks. Failure of the animals to quickly adapt to the formation of new memories may be analogous to the inflexibility in routine that is characteristic of autism. Numerous studies indicate that the HC is involved in learning and storing spatial information (Gage and Björklund, 1986; Morris et al., 1989; O’Keefe and Burgess, 1996). Our results showed that VPA rats have reduced GABAergic GAD67 expression in the HC that results in a hippocampal excitation-inhibition imbalance, and thus impaired network formation, which leads to abnormal learning and storage of information related to navigation. It should be mentioned that daily handling animals could lead to long-lasting effects on the brain and behavior. Growing evidence has showed that early handling increases the anxiety levels in adolescent rats (Jin et al., 2018). In this study, we cannot exclude the possibility that VPA-treated rats display to a larger extent of anxiety expression to decrease spatial learning than control rats, at least in part, due to early handing. Taken together, our findings show that prenatal exposure to VPA alters hippocampal and prefrontal GABAergic signaling, leading to the deficits in spatial learning and memory, as well as social functioning.

In summary, our data indicate that VPA-treated offspring rat produces behavioral and cognitive deficits that coexist with discrete alterations in excitatory-inhibitory balance in multiple brain regions, similar to those seen in individuals with autism. Moreover, although the exact mechanisms contributing to these deficits remain to be fully understood, our study provides strong evidence that pharmacologically enhancing GABA signaling could be a promising strategy for the treatment of autism.

## Author Contributions

QH, FY and SW were responsible for designing and organizing the project; prepared the manuscript. QH, YW, YL, DC, FY and SW performed the experiments and analyzed the data. All of the authors discussed the results and provided commentary on the manuscript.

## Conflict of Interest Statement

The authors declare that the research was conducted in the absence of any commercial or financial relationships that could be construed as a potential conflict of interest.
